# FOBI: an ontology to represent food intake data and associate it with metabolomic data

**DOI:** 10.1093/databa/baaa033

**Published:** 2020-06-17

**Authors:** Pol Castellano-Escuder, Raúl González-Domínguez, David S Wishart, Cristina Andrés-Lacueva, Alex Sánchez-Pla

**Affiliations:** 1 Biomarkers and Nutritional & Food Metabolomics Research Group, Department of Nutrition, Food Science and Gastronomy, University of Barcelona, Barcelona, Spain; 2 Statistics and Bioinformatics Research Group, Department of Genetics, Microbiology and Statistics, University of Barcelona, Barcelona, Spain; 3 CIBERFES, Instituto de Salud Carlos III, Madrid, Spain; 4 Department of Biological Sciences, University of Alberta, Edmonton, AB, T6G 2E8, Canada

## Abstract

Nutrition research can be conducted by using two complementary approaches: (i) traditional self-reporting methods or (ii) via metabolomics techniques to analyze food intake biomarkers in biofluids. However, the complexity and heterogeneity of these two very different types of data often hinder their analysis and integration. To manage this challenge, we have developed a novel ontology that describes food and their associated metabolite entities in a hierarchical way. This ontology uses a formal naming system, category definitions, properties and relations between both types of data. The ontology presented is called FOBI (Food-Biomarker Ontology) and it is composed of two interconnected sub-ontologies. One is a ’Food Ontology’ consisting of raw foods and ‘multi-component foods’ while the second is a ‘Biomarker Ontology’ containing food intake biomarkers classified by their chemical classes. These two sub-ontologies are conceptually independent but interconnected by different properties. This allows data and information regarding foods and food biomarkers to be visualized in a bidirectional way, going from metabolomics to nutritional data or vice versa. Potential applications of this ontology include the annotation of foods and biomarkers using a well-defined and consistent nomenclature, the standardized reporting of metabolomics workflows (e.g. metabolite identification, experimental design) or the application of different enrichment analysis approaches to analyze nutrimetabolomic data. **Availability**: FOBI is freely available in both OWL (Web Ontology Language) and OBO (Open Biomedical Ontologies) formats at the project’s Github repository (https://github.com/pcastellanoescuder/FoodBiomarkerOntology) and FOBI visualization tool is available in https://polcastellano.shinyapps.io/FOBI_Visualization_Tool/.

## 1 Introduction

The growing emergence of high-throughput analytical techniques in the life sciences over the past three decades, such as next-generation DNA sequencing, proteomics, metabolomics and other high-throughput omics approaches, has created significant challenges in data management. Currently, one of the main problems that researchers face lies in the question: *where are these data sets and how can I use them?* Unfortunately, the heterogeneity of storage platforms, data formats and privacy requirements of some of them often hinders their widespread access and use. In this vein, the creation of ontologies, defined as the ‘specification of a representational vocabulary for a shared domain of discourse—definitions of classes, relations, functions and other objects’ [1], is of vital importance to help analyze, annotate and homogenize these large and complex data sets [2, 3]. This is a major issue within the ‘FAIR Guiding Principles for scientific data management and stewardship’ [4], which aim to improve the findability, accessibility, interoperability and reusability of data. In particular, ontologies play a central role in the ‘Interoperability’ concept [1, 5], which establishes that ‘(meta)data has to use a formal, accessible, shared and broadly applicable language for knowledge representation’ [4].

Nutritional research largely relies on accurate dietary assessment, which is of great relevance to evaluate food intake and dietary habits. Dietary assessments also help in understanding the association between nutrition and health status. Nutritional research is often conducted by using two complementary approaches: (i) self-reporting methods (e.g. food frequency questionnaires, dietary recalls) [6] and (ii) the measurement of dietary biomarkers using a variety of analytical chemistry techniques, including metabolomics [7, 8]. With regard to traditional dietary assessment tools, it should be noted that subjective self-reports generate very complex textual data, containing types and quantities of foods and recipes in very diverse and heterogeneous formats that depend on the country/region, socio-demographic factors, etc.

To properly annotate this nutritional data using a common language, the most relevant ontology in nutrition research is FoodOn [9]. FoodOn is a comprehensive ontology composed of ‘term hierarchy facets’ that cover basic raw food source ingredients, packaging methods, cooking methods and preservation methods. It also includes an upper-level consisting of a variety of product type schemes under which food products can be categorized. On the other hand, the metabolomics standards initiative has also highlighted the importance of ontologies in metabolomics [10]. As Schlegel *et al*. reported, ‘the application of ontologies to metabolomics can improve the consistency of study data and can help link data using relationships that extend the computational capacity of the study data and enrich that knowledge source with a myriad of nationally available data to help fuel hypothesis driven laboratory based research’ [3].

In response to this, ChEBI (Chemical Entities of Biological Interest, https://www.ebi.ac.uk/chebi/) has developed a reference ontology for describing chemical compounds of biological interest in terms of their chemical structures, chemical categories and roles [11]. The ChEBI ontology is manually maintained and annotated. More recently, an automatic method for describing and classifying chemicals, called ClassyFire [12], has been developed and widely adopted by databases such as ChEBI, PubChem [13] and the Human Metabolome Database (HMDB) [14]. ClassyFire uses the ChemOnt ontology, consisting of more than 4800 different categories (with definitions) hierarchically structured into 11 different levels (Kingdom, SuperClass, Class, SubClass, etc.). Additionally, the HMDB has developed the ChemFOnt (chemical functional ontology) to describe the biological and industrial functions of all the compounds and metabolites found in this database. ChemFOnt consists of four major categories (physiological effect, disposition, process and role), 152 sub-categories and more than 4100 defined terms.

Although most existing ontologies have been specifically designed for a single theme, there are also some others composed of interconnected sub-ontologies, thus enabling users to establish relationships among different variables. For instance, ChEBI is organized in two sub-ontologies: (i) ‘Molecular Structure’, in which molecular entities are classified according to structure and (ii) ‘Subatomic Particle’, which classifies particles smaller than atoms. On the other hand, the Gene Ontology, includes three independent sub-ontologies: (i) ‘Biological process’, referred to a biological objective to which the gene or gene product contributes; (ii) ‘Molecular function’, defined as the biochemical activity of a gene product; and (iii) ‘Cellular component’, which refers to the place in the cell where a gene product is active [15]. In this regard, we would argue that nutritional research also generates large amounts of complex and inter-related data coming from self-reporting methods and metabolomics experiments. Therefore, an interconnected set of sub-ontologies would be particularly useful for defining relationships between both metabolomics data and self-reported dietary questionnaires. To facilitate the construction of such an ontology that describes both foods and their associated metabolite biomarkers, we will draw from several open-access databases. These include Exposome-Explorer [16], Phenol-Explorer [17], PhytoHub (http://phytohub.eu/) and Food Database (FooDB) (http://foodb.ca/)—all of which contain rich information about food constituents and food metabolites.

However, relationships between foods and their metabolites are extremely complex and the way they are described varies tremendously across these databases. This lack of commonality and the lack of a common, hierarchical structure makes data comparison and data searching quite difficult. Therefore, the development of a comprehensive ontology to clearly define the relationships between nutritional (food composition) and metabolomics (food metabolite or biomarker) data is needed. This ontology could have multiple practical applications in nutrimetabolomics, being the annotation of terms using a consistent and standardized nomenclature the most basic one, but of great importance in this research field due to the inherent complexity and heterogeneity of the data managed (i.e. multiple names/synonyms to define the same food/metabolite). Additionally, other potential applications of the ontology could be the ability to perform different enrichment analysis (e.g. to investigate patterns of food consumption on the basis of metabolomics data sets) or to conduct semantic similarity analysis (e.g. to establish novel associations between foods and metabolites). In this work, we describe FOBI (the Food-Biomarker Ontology), an ontology created with the aim of providing a common language to describe the many complex relationships in nutrimetabolomics research. This new ontology will allow users (and online databases) to integrate dictionaries and analyze these two kinds of data independently or together in a consistent and homogeneous way.

## 2 Results

FOBI is a freely available comprehensive ontology composed of two interconnected sub-ontologies including the ‘Food Ontology’ and the ‘Biomarker Ontology’. This ontology has been built using Protégé [18] and is available in OWL (Web Ontology Language) and OBO (Open Biomedical Ontologies) formats at the project’s Github repository (https://github.com/pcastellanoescuder/FoodBiomarkerOntology). FOBI consists of 1197 terms, 4 different properties, 13 food top-level classes, 11 biomarker top-level classes and more than 4500 relationships. Furthermore, FOBI is part of OBOFoundry project and FOBI IDs have been indexed into the HMDB and FooDB databases to facilitate the interoperability and the exchange of data.

### 2.1 Food Ontology

The Food Ontology was created on the basis of dietary data obtained from self-reported surveys for dietary assessment, including food frequency questionnaires (FFQ) and dietary recalls (DRs) [6]. The FFQ is a closed-ended survey that provides information on long-term dietary habits regarding a pre-defined list of 100–150 food items. On the other hand, DRs collect detailed information about foods consumed over a specific period (e.g. 24 hours, 3 days). To expand our Food Ontology as much as possible, we used the knowhow of our research group in working with FFQs and DRs collected from previous and ongoing projects. These projects involved cohorts from various European countries (e.g. Spain, France, United Kingdom). This allowed us to cover common foods for various dietary patterns, thus potentiating the applicability of FOBI in diverse research projects.

Accordingly, the Food Ontology is composed of more than 350 entities classified in different food classes. For this purpose, we considered both ‘raw foods’ and ‘multi-component foods’, with a multi-component food defined as any food item composed by two or more raw foods. In turn, the Food Ontology also describes the major ingredients forming part of each multi-component food according to the literature [19, 20]. These entities were annotated using a common nomenclature to reduce the complexity and heterogeneity of dietary data collected from free text questionnaires. This is because the same food/multi-component food can be named in many different ways (e.g. hamburger, burger, beef burger, etc.). Furthermore, FOBI also includes the FoodOn IDs for those food items common for both ontologies.

Major food classes in the Food Ontology were created considering both the nature of the food and the availability of food intake biomarkers for each class. A total of 13 food top-level classes were generated: beverage food product, cacao food product, dairy food product, egg food product, flavouring additive, fruits and vegetables, grain plant, lipid food product, meat food product, multi-component food, nuts and legumes, spice or herb and sugar. In turn, each of these 13 top-level classes have different subclass structures depending on its nature.

### 2.2 Biomarker Ontology

Food intake biomarkers (FIBs) are compounds derived directly from foods or the metabolism of food compounds that are characteristic or particular to a specific food item (e.g. phloretin for apple) or food category (e.g. glucosinolates for cruciferous vegetables) [7]. An important aspect to highlight on this regard is that, although the concentration of these metabolites in the food product may vary as a response to different factors (e.g. variety, agronomic practices, breeding, food processing), FIBs can always be associated with the consumption of the corresponding food (i.e. apple always contains phloretin, regardless the variety or cultivation conditions). FIBs potentially consist of a vast number of chemicals with very different physico-chemical properties, including polyphenols and carotenoids, coming from plant-derived foods; derivatives of amino acids and fatty acids (mainly found in animal products); methylxanthines from coffee, tea and cocoa; alkaloids, organic acids and many others. Food constituents can undergo multiple biotransformation steps after ingestion, thus significantly expanding their metabolic complexity. Typically, xenobiotic food constituents are first subjected to phase I and phase II transformations, principally in the liver, kidneys and intestine, for detoxification purposes and to facilitate their excretion. Phase I metabolism normally involves cytochrome P450-mediated oxidation and hydrolysis transformations, while phase II reactions consist of chemical conjugations, such as methylation, acetylation, sulfation, glucuronidation and amino acid conjugation [21]. The gut microbiota also plays a major role in the metabolism of poorly bioavailable food derived metabolites, usually involving ring cleavage reactions and a variety of fermentative pathways to produce smaller, more easily absorbed derivatives [22]. Rather than trying to handle all possible compounds (possibly numbering in the tens of thousands), we chose to gather currently reported food derived metabolites and to define their relationships with foods and dietary patterns.

To create the Biomarker Ontology, we considered almost 600 known food metabolites, including dietary compounds and their host and microbiota-derived metabolites. These compounds were compiled from extensive literature reviews and the information contained in open access databases such as Phenol-Explorer, PhytoHUB and the FooDB. Of particular help was the material produced by the EU-funded FoodBAll project (http://foodmetabolome.org/), which worked on discovering and validating FIBs for a range of foods. The FoodBAll consortium has produced a collection of review articles published over the past 2 years focused on the most frequently consumed food groups [23–30]. Supplementary Table S1 summarizes the major classes of FIBs included in our first draft of FOBI and their associations with foods. It should be noted that this sub-ontology is only composed by food derived metabolites, while biomarkers of effect (i.e. endogenous metabolites altered after food intake) have been discarded. This is not intended to be a final, definitive ontology of food intake biomarkers, since it will be updated with novel FIBs as new studies are reported.

The FIBs in the Biomarker Ontology were classified according to their chemical classes using ClassyFire [12] and ChemOnt (version 2.1).

A key challenge in creating this sub-ontology was the complexity and diversity of the chemical nomenclature of food derived metabolites. For instance, caffeic acid, a relatively simple phenolic acid found in numerous foods such as coffee, can also be named as (E)-3-(3,4-dihydroxyphenyl)prop-2-enoic acid (IUPAC name), trans-3,4-dihydroxycinnamic acid, trans-3,4-dihydroxycinnamate or 3-(3,4-dihydroxyphenyl)acrylic acid, among other names. This disparity is even greater for more complex metabolites or phase II derivatives (e.g. caffeic acid 3-glucuronide, 3,4-dihydroxycinnamic acid 3-glucuronide, 4-hydroxycinnamic acid 3-O-glucuronide, (2S,3S,4S,5R,6S)-6-5-[(1E)-2-carboxyeth-1-en-1-yl]-2-hydroxyphenoxy-3,4,5-trihydroxyoxane-2-carboxylate). To facilitate the use of FOBI, metabolites are named according to the nomenclature commonly employed by nutrimetabolomic researchers, which easily enables users to differentiate isomers and similar metabolites within the same chemical class. Besides the FOBI ID, this ontology also lists the code numbers for HMDB, KEGG, ChEBI, PubChem, InChIKey, InChI and ChemSpider for all these compounds, if available, which further facilitates the interoperability of FOBI and the exchange of data. In addition, the Biomarker Ontology also contains some putative FIBs previously identified via targeted metabolomics by our research group [31, 32]. It should be noted that, for most of these biomarkers, only InChIKey and InChI codes are available. This is because only a few of them are listed in HMBD, KEGG, ChEBI, PubChem or ChemSpider so there is limited information about their biological roles and potential food sources.

In addition, we have created a synonym file with all these annotations for all food intake biomarkers or food metabolites included in the Biomarker Ontology, which can be freely download as a.csv file (Supplementary Table S2).

### 2.3 Ontology architecture

The architecture of FOBI is composed by classes corresponding to the items from the two sub-ontologies previously described (Food and Biomarker Ontologies), based on ChEBI (for metabolites) and FoodOn (for foods), respectively, and edges representing their relationships. Within the Food Ontology, raw foods are connected with the corresponding food class by the property *is_a*. On the other hand, multi-component foods are related to raw foods by the property *Contains*, in the same way that raw foods are connected with multi-component foods in the form of *IsIngredientOf*. For the Biomarker Ontology, the relationship between individual metabolites and the chemical class (defined by ClassyFire) is also defined by the property *is_a*. Finally, nodes from the Food and Biomarker Ontologies are interconnected by the inverse properties *BiomarkerOf* and *HasBiomarker*.

**Figure 1:**
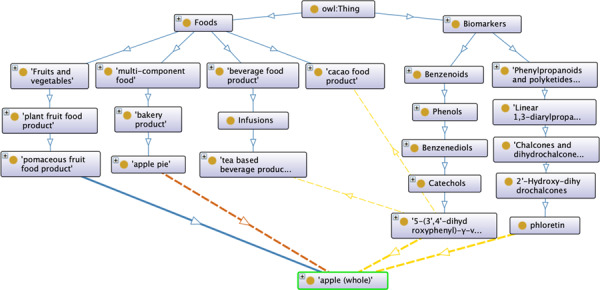
FOBI architecture considering apple as an example.

Figure 1 illustrates the FOBI architecture considering apple as an example. According to this, apple can be a raw food with the following relationships ‘apple *is_a* pomaceous fruit food product *is_a* plant fruit food product *is_a* Fruits and vegetables *is_a* Food’ (the property *is_a* is represented by blue arrows). In addition, apple can also be an ingredient in multi-component foods such as apple pie, so that ‘apple *IsIngredientOf* apple pie *is_a* bakery product *is_a* multi-component food *is_a* Food’ as well ‘apple pie *Contains* apple’ (the properties *IsIngredientOf* and *Contains* are represented by orange arrows). Considering phloretin and 5-(3’,4’-dihydroxyphenyl)-$\gamma$-valerolactone as biomarkers of apple intake, they can be categorized as ‘phloretin *is_a* 2’-Hydroxy-dihydrochalcone *is_a* Chalcones and dihydrochalcones *is_a* Linear 1,3-diarylpropanoid *is_a* Phenylpropanoids and polyketides *is_a* Biomarker’ and ‘5-(3’,4’-dihydroxyphenyl)-$\gamma$-valerolactone *is_a* Catechol *is_a* Benzenediol *is_a* Phenol *is_a* Benzenoid *is_a* Biomarker’. Because phloretin is a specific marker of apple, this metabolite is exclusively connected via the Food Ontology by the relationships ‘phloretin *BiomarkerOf* apple’ and ‘apple *HasBiomarker* phloretin’ (the properties *BiomarkerOf* and *HasBiomarker* are represented by yellow arrows). On the other hand, 5-(3’,4’-dihydroxyphenyl)-$\gamma$-valerolactone can be derived from various procyanidin-rich foods (cacao, tea), so it can be connected with them following the same structure described for apple.

### 2.4 FOBI network analysis

To evaluate the information content of FOBI and its efficiency, we conducted network analysis to compute the average path length (APL) among FOBI’s network nodes. The APL is defined as the average number of steps along the shortest paths for all possible pairs of network nodes. The APL can be used for enrichment analysis [33] and is considered a robust measure of a network’s topology and its efficiency of information transport [34]. From a more pragmatic point view, the APL can be thought of as a measure to demonstrate whether the entities (or nodes) within an ontology are functionally cohesive. Thus, nodes with high cohesive functionality tend to have lower APL values compared to randomly selected nodes [33].

If we consider an unweighted directed graph $G$ with the set of vertices $V$. Let $d(v_{1},v_{2})$, where $v_{1},v_{2}\in V$ denote the shortest distance between $v_{1}$ and $v_{2}$. Assume that $d(v_{1},v_{2})=0$ if $v_{2}$ cannot be reached from $v_{1}$. Then, the APL $l_{G}$ is:

(1) \begin{equation*}l_{G}={\frac{1}{n\cdot (n-1)}}\cdot \sum _{i\neq j}d(v_{i},v_{j}),\end{equation*}

where $n$ is the number of vertices in $G$.

**Figure 2:**
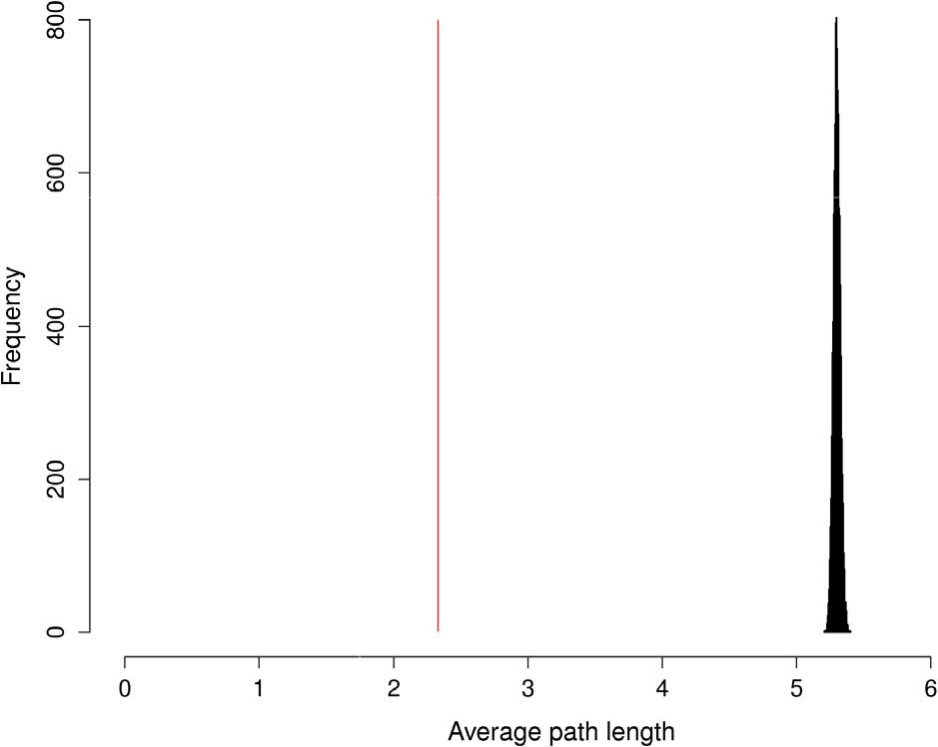
APL of FOBI versus random graphs APLs.

To evaluate the FOBI network, we first calculated its APL and then created 10 000 random graphs using the Erdös–Rényi algorithm [35] and calculated the mean of their APLs. The computed FOBI APL value was 2.33, which is 114.26 standard deviations below the random mean APL (5.30) (Figure 2), thus demonstrating the very high information transport efficiency of FOBI compared to a random network.

### 2.5 Implementation of the FOBI web application

The FOBI’s web application (https://polcastellano.shinyapps.io/FOBI_Visualization_Tool/) is powered by Shiny (https://shiny.rstudio.com). This Shiny app imports all FOBI relationships in R and organize them in a table or in a graph according to the user input.

**Figure 3:**
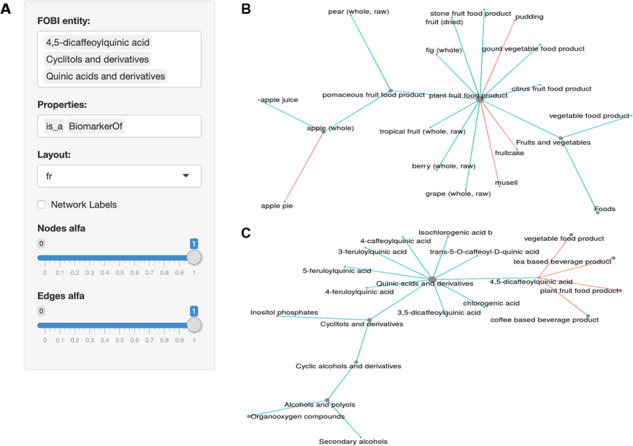
The FOBI web application. (**A**) Settings panel of the web interface; (**B**) interrelationships of food items in FOBI; (**C**) relationships between food items and metabolites in FOBI.

The FOBI application settings panel is shown in Figure 3A. This user-friendly application accepts both food and biomarker entries as FOBI entity, which can be displayed using multiple layouts and using different properties (*is_a*, *BiomarkerOf* and *Contains*). Results can be downloaded in either a table or a graph format.

As summarized in Figure 3, this FOBI application considerably simplifies inspection of the interrelationships between foods and biomarkers. On one hand, food items can be interrelated by their properties *is_a* and *Contains* to show their classification according to food classes and the presence of ingredients in complex multi-component foods (Figure 3B). Similarly, food intake biomarkers can also be categorized according to their chemical class and additionally related to foods by their properties *is_a* and *BiomarkerOf* (Figure 3C).

## 3 Discussion

Knowledge of the complex inter-relationships between food types, food components, food ingredients and food intake biomarkers is critical to facilitate our understanding of nutrition and metabolism. Such an understanding will enable accurate metabolomics-based food intake assessment and assist with the development of personalized nutrition strategies to select specific diets according to the subject’s phenotype, disease state, microbiome, metabolome, etc.

To this end, we have developed the FOBI. This is a nutrition-specific ontology composed of two sub-ontologies with an independent hierarchy but clear relationships between them. The Food Ontology consists of known foods grouped according to their major (13) nutritional classes, while the Biomarker Ontology contains food derived metabolites categorized according to their chemical classes. The edges linking these two sub-ontologies define, using a common language, the hierarchy of each food and food biomarker entity, as well as the properties that relate these two kinds of data. This architecture can be easily interpreted at both the user and computational level. Likewise, FOBI can be used for different purposes, from making simple queries to complex computational queries simultaneously using all the information stored in the ontology.

Ontologies facilitate many practical applications, such as annotating entities or items, performing enrichment analysis (e.g. over representation analysis), conducting semantic similarity analysis [2] or even to find unexpected patterns. Some of the potential applications of FOBI are described below. The most basic application of FOBI is in the annotation of foods and related food biomarkers using a consistent, well-defined, fully standardized nomenclature. This will facilitate the comparability and interoperability between nutrition studies, projects and research groups. FOBI will also facilitate nutrimetabolomics research thanks to the comprehensive description of associations between food types and food derived metabolites, as summarized in Supplementary Table S1. For instance, interrelationships defined in this ontology, together with the accurate nomenclature defined in the synonym file, will be particularly useful in untargeted metabolomics studies (e.g. acute intervention studies) for discriminant feature identification.

Furthermore, this information can also serve to facilitate study designs, from hypothesis generation (e.g. expected metabolites occurring after a dietary intervention) to experimental design (e.g. optimization of targeted metabolomics methods focused on analytes of interest). Additionally, the availability of FOBI will give nutrimetabolomic researchers the ability to perform enrichment analysis. Given a set of metabolites (e.g. discriminant metabolites identified in a metabolomics study), the hierarchical structure of FOBI enables one to evaluate possible over-representation of specific chemical classes, which could reflect the consumption of particular foods or food groups. This could be a first step towards ‘food enrichment analysis’.

### 3.1 Limitations

FOBI has two main limitations. The first one concerns the relationship between foods and their metabolites. The relationships that FOBI contain are limited to the best known and most frequent. However, there can be relationships between foods and their metabolites that FOBI does not contain due to the fact that not every food compound (or its metabolite) has been tested for its presence in certain foods or within certain human biofluids.

The second limitation concerns the limited number of foods and food metabolites/biomarkers in FOBI. Currently, FOBI has more than 350 food nodes (in total) and 590 food biomarkers (only metabolites) corresponding to more than 4500 relationships (among foods, among biomarkers and between foods and biomarkers). As with most ontologies, FOBI is undergoing constant evolution and development. As a result, the number of entities and the quality of relationships described in this ontology will be continuously increasing and improving.

### 3.2 Future work

FOBI is an open-source project that can be readily used and enhanced by anyone in the nutritional and nutrimetabolomic community. Further expansion of the ontology to cover more food types, more food biomarkers and more relationships will certainly increase its utility.

Future efforts will be directed at expanding this ontology and extending it so that it is more widely used in other curated databases such as Exposome-Explorer, Phenol-Explorer, HMDB and FooDB.

## 4 Conclusion

FOBI is the first ontology that integrates nutritional and metabolomic data in a comprehensive common language. At the moment, FOBI has a total of 1197 terms (366 from Food Ontology and 831 from Biomarker Ontology), 11 chemical top-level classes, 13 food top-level classes and 4 different properties that are fully defined and which have clear relationship mappings. FOBI defines the relationships between foods and their metabolites (biomarkers) through a formal ontology.

FOBI allows experts to annotate and analyze nutritional and metabolomic data in a consistent way, making the results comparable between and across studies in the same field. The development of FOBI will lead to an improvement in the interoperability of nutritional and nutrimetabolomic data thereby making the data sets generated from these studies fully FAIR compliant.

## Supplementary Material

Supplementary_Tables_baaa033

## References

[1] Kramer F. and Beißbarth T. (2017) Working with ontologies. Methods Mol. Biol., 1525, 123–135. 27896720 10.1007/978-1-4939-6622-6_6

[2] Hoehndorf R., Schofield P.N. and Gkoutos G.V. (2015) The role of ontologies in biological and biomedical research: a functional perspective. Brief. Bioinform., 16, 1069–1080. 25863278 10.1093/bib/bbv011 PMC4652617

[3] Schlegel D.R., Ruttenberg A. and Elkin P.L. (2015) Ontologies in Metabolomics. Metabolomics, 5, e137.

[4] Wilkinson M.D., Dumontier M., Aalbersberg I.J. et al. (2016) The FAIR Guiding Principles for scientific data management and stewardship. Sci. Data, 3, 160018. 26978244 10.1038/sdata.2016.18 PMC4792175

[5] Noy N.F. and McGuinness D.L. (2001) Ontology development 101: a guide to creating your first ontology. Stanford Knowledge Systems Laboratory Technical Report, KSL-01-05 and Stanford Medical Informatics Technical Report, SMI-2001-0880.

[6] Shim J.S., Oh K. and Kim H.C. (2014) Dietary assessment methods in epidemiologic studies. Epidemiol. Health, 36. 10.4178/epih/e2014009 PMC4154347 25078382

[7] Scalbert A., Brennan L., Manach C. et al. (2014) The food metabolome: a window over dietary exposure. Am. J. Clin. Nutr., 99, 1286–1308. 24760973 10.3945/ajcn.113.076133

[8] Ulaszewska M.M., Weinert C.H., Trimigno A. et al. (2019) Nutrimetabolomics: an integrative action for metabolomic analyses in human nutritional studies. Mol. Nutr. Food Res., 63, 1800384. 10.1002/mnfr.201800384 30176196

[9] Dooley D.M., Griffiths E.J., Gosal G.S. et al. (2018) FoodOn: a harmonized food ontology to increase global food traceability, quality control and data integration. NPJ Sci. Food, 2, 23. 31304272 10.1038/s41538-018-0032-6 PMC6550238

[10] Sansone S.A., Schober D., Atherton H.J. et al. (2007) Metabolomics standards initiative: ontology working group work in progress. Metabolomics, 3, 249–256.

[11] Degtyarenko K., De Matos P., Ennis M. et al. (2007) ChEBI: a database and ontology for chemical entities of biological interest. Nucleic Acids Res., 36, D344–D350. 17932057 10.1093/nar/gkm791 PMC2238832

[12] Feunang Y.D., Eisner R., Knox C. et al. (2016) ClassyFire: automated chemical classification with a comprehensive, computable taxonomy. J. Cheminform., 8, 61. 27867422 10.1186/s13321-016-0174-y PMC5096306

[13] Kim S., Chen J., Cheng T. et al. (2019) PubChem 2019 update: improved access to chemical data. Nucleic Acids Res., 47, D1102–D1109. 30371825 10.1093/nar/gky1033 PMC6324075

[14] Wishart D.S., Feunang Y.D., Marcu A. et al. (2017) HMDB 4.0: the human metabolome database for 2018. Nucleic Acids Res., 46, D608–D617. 10.1093/nar/gkx1089 PMC5753273 29140435

[15] Ashburner M., Ball C.A., Blake J.A. et al. (2000) Gene ontology: tool for the unification of biology. Nat. Genet., 25, 25–29. 10802651 10.1038/75556 PMC3037419

[16] Neveu V., Moussy A., Rouaix H. et al. (2016) Exposome-Explorer: a manually-curated database on biomarkers of exposure to dietary and environmental factors. Nucleic Acids Res., 45, D979–D984. 27924041 10.1093/nar/gkw980 PMC5210656

[17] Rothwell J.A., Perez-Jimenez J., Neveu V. et al. (2013) Phenol-Explorer 3.0: a major update of the Phenol-Explorer database to incorporate data on the effects of food processing on polyphenol content. Database, 2013:bat070. 24103452 10.1093/database/bat070 PMC3792339

[18] Musen M.A. (2015) The protégé project: a look back and a look forward. AI Matters, 1, 4–12. 27239556 10.1145/2757001.2757003 PMC4883684

[19] McCance R.A. and Widdowson E.M. (2014) The Composition of Foods. Royal Society of Chemistry, Cambridge, UK.

[20] Reinivuo H., Bell S. and Ovaskainen M.L. (2009) Harmonisation of recipe calculation procedures in European food composition databases. J. Food Compos. Anal., 22, 410–413.

[21] Manach C. and Donovan J.L. (2004) Pharmacokinetics and metabolism of dietary flavonoids in humans. Free Radic. Res., 38, 771–785. 15493450 10.1080/10715760410001727858

[22] Rowland I., Gibson G., Heinken A. et al. (2018) Gut microbiota functions: metabolism of nutrients and other food components. Eur. J. Nutr., 57, 1–24. 10.1007/s00394-017-1445-8 PMC5847071 28393285

[23] Rothwell J.A., Madrid-Gambin F., Garcia-Aloy M. et al. (2018) Biomarkers of intake for coffee, tea, and sweetened beverages. Genes Nutr., 13, 15. 29997698 10.1186/s12263-018-0607-5 PMC6030755

[24] Michielsen C.C., Almanza-Aguilera E., Brouwer-Brolsma E.M. et al. (2018) Biomarkers of food intake for cocoa and liquorice (products): a systematic review. Genes Nutr., 13, 22. 30065791 10.1186/s12263-018-0610-x PMC6062926

[25] Praticò G., Gao Q., Manach C. et al. (2018) Biomarkers of food intake for Allium vegetables. Genes Nutr., 13, 34. 30607216 10.1186/s12263-018-0624-4 PMC6309086

[26] Ulaszewska M., Vázquez-Manjarrez N., Garcia-Aloy M. et al. (2018) Food intake biomarkers for apple, pear, and stone fruit. Genes Nutr., 13, 29. 30519365 10.1186/s12263-018-0620-8 PMC6267079

[27] Münger L.H., Garcia-Aloy M., Vázquez-Fresno R. et al. (2018) Biomarker of food intake for assessing the consumption of dairy and egg products. Genes Nutr., 13, 26. 30279743 10.1186/s12263-018-0615-5 PMC6162878

[28] Zhou X., Gao Q., Praticò G. et al. (2019) Biomarkers of tuber intake. Genes Nutr., 14, 9. 30984301 10.1186/s12263-019-0631-0 PMC6444566

[29] Garcia-Aloy M., Hulshof P.J., Estruel-Amades S. et al. (2019) Biomarkers of food intake for nuts and vegetable oils: an extensive literature search. Genes Nutr., 14, 7. 30923582 10.1186/s12263-019-0628-8 PMC6423890

[30] Harsha P.S.S., Wahab R.A., Garcia-Aloy M. et al. (2018) Biomarkers of legume intake in human intervention and observational studies: a systematic review. Genes Nutr., 13, 25. 30214640 10.1186/s12263-018-0614-6 PMC6131749

[31] González-Domínguez R., Urpi-Sarda M., Jáuregui O. et al. (2020) Quantitative dietary fingerprinting (QDF)—a novel tool for comprehensive dietary assessment based on urinary nutrimetabolomics. J. Agric. Food Chem., 68, 1851–1861. 30799616 10.1021/acs.jafc.8b07023

[32] González-Domínguez R., Jáuregui O., Mena P. et al. (2020) Quantifying the human diet in the crosstalk between nutrition and health by multi-targeted metabolomics of food and microbiota-derived metabolites. Int. J. Obes., In press, 10.1038/s41366-020-0628-1 32541919

[33] Embar V., Handen A. and Ganapathiraju M.K. (2016) Is the average shortest path length of gene set a reflection of their biological relatedness? J. Bioinform. Comput. Biol., 14, 1660002. 28073302 10.1142/S0219720016600027 PMC5726383

[34] Albert R. and Barabási A.L. (2002) Statistical mechanics of complex networks. Rev. Mod. Phys., 74, 47.

[35] Erdös P. and Rényi A. (1959) On random graphs. Publ. Math. Debrecen, 6, 290–297.

